# Training the next generation of community-engaged physicians: a mixed-methods evaluation of a novel course for medical service learning in the COVID-19 era

**DOI:** 10.1186/s12909-024-05372-8

**Published:** 2024-04-22

**Authors:** Jack J. Scala, Hannah Cha, Kiarash Shamardani, Emma R. Rashes, Lehi Acosta-Alvarez, Rishi P. Mediratta

**Affiliations:** 1 Department of Biology, BS Candidate, Stanford University, Palo Alto, CA USA; 2 Department of Symbolic Systems, BS Candidate, Stanford University, Palo Alto, CA USA; 3 Stanford University School of Medicine, Palo Alto, CA USA; 4 Faculty of Medicine, Tel Aviv University, Tel Aviv, Israel; 5 Department of Pediatrics, Division of Pediatric Hospital Medicine, Stanford University School of Medicine, Palo Alto, CA USA

**Keywords:** Medical service learning, Community-engaged learning, Self-determination theory, Public health work, Motivation, Student autonomy

## Abstract

**Background:**

Medical school curricula strive to train community-engaged and culturally competent physicians, and many use service learning to instill these values in students. The current standards for medical service learning frameworks have opportunities for improvement, such as encouraging students to have more sustainable and reciprocal impact and to ingrain service learning as a value to carry throughout their careers rather than a one-time experience. PEDS 220: A COVID-19 Elective is a Stanford University course on the frontlines of this shift; it provides timely education on the COVID-19 pandemic, integrating community-oriented public health work to help mitigate its impact.

**Methods:**

To analyze our medical service learning curriculum, we combined qualitative and quantitative methods to understand our students’ experiences. Participants completed the Course Experience Questionnaire via Qualtrics, and were invited to complete an additional interview via Zoom. Interview transcripts were analyzed using an interactive, inductive, and team-based codebook development process, where recurring themes were identified across participant interviews.

**Results:**

We demonstrate through self-determination theory that our novel curriculum gives students valuable leadership and project management experience, awards strong academic and community-based connections, and motivates them to pursue future community-engaged work.

**Conclusions:**

This educational framework, revolving around students, communities, and diversity, can be used beyond the COVID-19 pandemic at other educational institutions to teach students how to solve other emergent global health problems. Using proven strategies that empower future physicians to view interdisciplinary, community-engaged work as a core pillar of their responsibility to their patients and communities ensures long-term, sustainable positive impact.

**Trial registration:**

N/A.

**Supplementary Information:**

The online version contains supplementary material available at 10.1186/s12909-024-05372-8.

## Background

In medical service learning, students actively address community health needs by engaging with the community and reflecting on their experiences upon returning to the classroom [1, 2]. Medical service learning has improved students’ empathy, interpersonal and leadership skills, and cultural competency and sensitivity, which are difficult characteristics to teach through traditional didactic methods [3–9]. Medical service learning, a component of community engagement, brings medical practitioners and the communities they serve closer together [10]. While this approach to experiential learning has been utilized to teach medical students, our definition of medical service learning includes undergraduate and graduate students from any discipline addressing a community health need. Through this model, students gain a more nuanced and complex understanding of the communities that they serve [11] Furthermore, self-determination theory (SDT), a framework that draws upon how heightened levels of autonomy, competence, and relatedness lead to increased levels of motivation, can be used to motivate medical students in community health work [12–14].

Medical service learning approaches vary in educational pedagogy, area of emphasis, and student educational level. An effective way to encourage students to participate in community health work involves incorporating medical service into coursework. This approach provides students with protected time to participate in community service, which they have cited as a barrier to participating in community health work [15, 16]. Other strategies allow students to volunteer their time outside of their structured coursework [4, 17, 18]. Some medical service learning courses focus on specific community health issues, including COVID-19, diabetes, and breast cancer [16, 19, 20], whereas others focus on support for vulnerable communities, such as immigrant farm workers [21]. Medical service learning, by virtue of its ability to connect academic medical institutions to any community, has also been effective in addressing immediate global health needs, such as disparities in prenatal care and tuberculosis outbreaks, as well as empowering the next generation of global health leaders through exposure to global health projects at early stages in students’ careers [22, 23]. Medical service learning can be applied to teaching throughout the medical education continuum, from pre-medical students to medical residents [16, 24–26].

Current medical service learning courses have limitations that hinder students’ development as leaders in solving community health problems. Predetermined roles limit students’ autonomy and contribute to high turnover because they may not feel a sense of personal responsibility for the project [27–29]. This limitation of student autonomy and relatedness to work, according to SDT, may lead to lessened motivation for community engagement. Additionally, these courses lack diversity and are taken by a homogenous cohort of students, such as first-year medical students [2, 3]. This represents a missed opportunity to train students to work with others from different professional backgrounds on community health issues, better preparing students to work in an interprofessional healthcare network.

To address these limitations, we developed the COVID-19 Elective course at the Stanford University School of Medicine [16]. The first goal of the course is to give students up-to-date information on the COVID-19 pandemic’s medical, public health, and social implications. The second goal of the course is to allow students to collaborate with community partners to creatively implement service learning projects that addresses COVID-19-related, community-based challenges, from ameliorating vaccine hesitancy to tackling misinformation. This study evaluates past students’ attitudes toward the curriculum in order to evaluate the course’s ability to serve as a gateway into the world of medical service learning. Our novel curriculum targets undergraduate, graduate, and medical students by providing an interdisciplinary method of training future community health leaders.

## Methods

### Study design

After the implementation of our medical service learning curriculum, we developed a mixed-methods study to understand students’ experience in the course and their attitudes toward future medical service opportunities. Mixed-methods studies integrate both qualitative and quantitative approaches providing a more comprehensive understanding of the research question. This design provides us with a broader perspective by examining relationships, patterns, and generalizability across a larger sample allowing us to both explore the depth of student experiences and behaviors and also generate statistical evidence to support the findings. Using quantitative methods, such as the course experience questionnaire (CEQ), we elucidated the skills students acquired from undertaking a self-driven medical service learning project. Using qualitative methods, we understood general attitudes towards the course and gauged students’ motivation for pursuing medical service opportunities. SDT was used as a conceptual framework for the qualitative methods. After extrapolating qualitative themes from interview transcripts, to bolster credibility, we conducted member checking. By sending the resultant themes to participants and providing the opportunity for feedback, we ensured the accuracy of the final themes. Taken together, these data will be used to assess the potential for the course to serve as a gateway to the world of medical service learning.

### Course curriculum

To provide undergraduate, graduate, and medical students with up-to-date information on the COVID-19 pandemic and allow students an opportunity to participate in developing creative solutions to problems that arose during the pandemic, we designed and implemented the COVID-19 Elective course in March 2020, using Kern’s Six Steps for curricular development [16]. As previously described, we centered our curriculum on collaboration, interdisciplinary values, and community engagement. The current strategies for community engagement implemented in medical school curriculums miss opportunities for long-term impact and engagement. Our curriculum encourages students to use their skills and collaborate with those in different fields to create innovative and longstanding interventions to advance community health. After each iteration of the course, we improved our curriculum based on student feedback [16]. All students attended weekly COVID-19-related seminars on a variety of topics, including social determinants of health, healthcare disparities, the clinical management of COVID-19, and vaccine equity. Students who chose to take the course for additional credit completed a community-based, COVID-19-related project by partnering with community partners. Community partners are community organizations or professionals, identified with the help of the course’s Teaching Team, with which students worked closely to learn more about the needs of the community they were working with and implement their projects. In addition to the weekly seminar on COVID-19, these students participated in a weekly discussion section focused on the best practices for community-engaged work. At the end of the academic term, students presented their projects at a symposium attended by their fellow students, community partners, and members of the broader Stanford University community. The goals of the community-engaged section are to provide an introduction to medical service learning in the context of COVID-19 and prepare students to serve as future leaders in medical service. We have guided 30 students in creating self-designed course projects over the past two years.

To resemble the multidisciplinary student population, which ranged from undergraduate students to practicing physicians, the Teaching Team included an attending physician, undergraduate students, and graduate students.

### Participants

Former COVID-19 Elective students who enrolled for additional credit and completed community-engaged projects were invited to participate in the study. Permission to conduct the study was granted by the ethics committee of our university, and all participants provided written informed consent prior to participating in the study. Participants were recruited to the study via an email sent to every former student who had completed a course project. The initial recruitment email provided students with a Qualtrics link to verify participant eligibility and obtain informed consent.

### Quantitative data collection

Data on students’ experience in the course and attitudes about their careers and community-engaged work were collected via a Course Experience Questionnaire (CEQ) and one-on-one interviews. CEQ has been used as a measure of perceived teaching quality in undergraduate and graduate degree medical education programs in Australia and the United Kingdom. The validity of CEQ is established by demonstrating positive correlations with students’ approaches to learning, perceived course satisfaction, academic achievement, and reported generic skills development.^29^

Out of 30 students who completed the course over a two-year timespan, 9 students consented to the study using a form approved by the Stanford University Institutional Review Board. These students asynchronously completed the CEQ via Qualtrics about their experience in the course. Students who participated in any part of the study received a $10 gift certificate.

The CEQ was administered after final grades were posted at the conclusion of the course. The validated 23-item CEQ was chosen for post-course assessment to evaluate student experiences with and perceptions of our teaching paradigm [30]. For each item, participants rated their level of agreement on a 5-point Likert scale (from ‘‘strongly disagree’’ to ‘‘strongly agree’’). Descriptive statistics were generated for each item of the CEQ and each of its scales evaluating student perception of the teaching paradigm using Microsoft Excel. Internal reliability of the scales (good teaching, generic skills, clear goals and standards, and graduate qualities) was measured by the Cronbach alpha coefficient.

### Student data collection

Among the 9 students who completed the CEQ, 5 students completed one hour-long one-on-one interviews (Table 1). Interviews were conducted via Zoom video conferencing; and video, audio, and text transcripts were recorded via the Zoom software. Zoom video conferencing was selected over in-person interviewing as many of our participants were no longer living in the area.

**Table 1:** Participants and project profiles from qualitative participants

	Educational level	Project
Participant 1 (P1)	Undergraduate student, Biology (pre-med)	Wrote and disseminated a children’s book to combat vaccine hesitancy in multiple languages
Participant 2 (P2)	Undergraduate student, Human Computer Interaction	Created an educational board game to teach children the importance of hygienic habits and vaccination
Participant 3 (P3)	Masters student, Computer Science	Developed an IRB proposal for the validation of an algorithm capable of detecting COVID-19 infection from a cough audio file using artificial intelligence
Participant 4 (P4)	Medical student	Collected and disseminated masks to student residences on a university campus
Participant 5 (P5)	Ph.D. student, Engineering	Created a needs-assessment for natural disaster evacuations during the COVID-19 pandemic to prevent outbreaks during natural disasters

To develop qualitative interview questions, a theoretical framework was developed based on SDT and the goals and objectives of the curriculum and previously established frameworks for studying the efficacy of service-learning curriculums [31, 32]. The areas identified in our theoretical framework included those that evaluate how the course addressed the needs of and benefited our students and their community partners. These areas included mentorship, project impact, career trajectory, and short and long-term goals, which were revealed through students’ attitudes (Tables 2 and 3). A semi-structured interview guide was developed based on this theoretical framework with the goal of understanding students’ attitudes about the areas identified in the framework (Table 2). Students were asked questions about their career goals, motivations for taking the course, experience with their community-engaged project, experience with mentorship in the course, and how their career goals and opinions on mentorship and community-engaged work have changed due to their participation in the course (Supplementary Digital Appendix 1). The semi-structured questions were piloted during a mock interview with a member of the research team who was a former student in the course. After this mock interview, the team modified our questions and developed additional questions based on gaps identified from the mock interview. After the interviews were complete, Zoom transcripts were corrected for spelling, grammar, and clarity.

**Table 2:** Theoretical frameworks that guided interview questions

	Student participant interview question	Referenced theoretical framework
1	Why did you decide to take PEDS 220: COVID-19 Elective?	SDT: Autonomy, Relatedness
2	Describe your experience with the course’s mentorship model.	Authenticity Through Service Learning
3	What was most helpful about the mentorship model? What can be improved?	Service Learning: Academic Learning
4	To what extent were you satisfied with the Teaching Team’s level of involvement in your project? If not, how would you have preferred it to be different?	SDT: Relatedness
5	How did completing a mentored, community-engaged project change your philosophy of education?	Service Learning: Academic Learning
6	Describe your career aspirations. Have they changed or evolved as a result of taking PEDS 220 and completing a community-engaged project?	SDT: Competence
7	Describe your educational aspirations. Have they changed or evolved as a result of taking PEDS 220 and completing a community-engaged project?	SDT: Competence
8	What were the biggest takeaways from your project?	SDT: Autonomy
9	How will completing a community-engaged and mentored project change how you will approach future scholarly projects?	Service Learning: Civic Learning
10	How did your course project impact your career trajectory in the short term?	Service Learning: Meaningful Community Service
11	How did your course project impact your career trajectory in the long term?	Service Learning: Meaningful Community Service
12	What did you learn about mentorship from taking this class?	Service Learning: Academic Learning
13	Did you find your community partnership to be effective in reaching the goals of your project?	SDT: Relatedness
14	Were you satisfied with your community partner’s level of involvement in your project? If not, how would you have preferred it to be different?	SDT: Relatedness Authenticity Through Service Learning

**Table 3:** Characteristics of course experience questionnaire scales used in study (*n* = 9)

Scale	Item	Mean	SD	Cronbach α
Good teaching	6	4.65	0.62	0.92
Generic skills	7	3.87	1.1	0.82
Clear goals and standards	3	4.41	1.08	0.76
Graduate qualities	6	4.39	0.96	0.79
Overall satisfaction	1	4.56	0.73	N/A

* N/A indicates not available

Table demonstrating how interview questions were derived from the following theoretical frameworks: Self-Determination Theory (SDT), Service Learning, and Authenticity Through Service Learning.

### Qualitative data analysis process

We conducted an iterative, inductive, and team- based codebook development process to generate themes from the interviews [33]. De-identified interview transcripts were analyzed using NVivo software (QSR International, Cambridge, MA), which involved initial and focused coding, code reconciliation, and the team-based creation of a codebook [33]. Through this software, our team created categorizations of participants’ experiences through iterative, inductive-based codebook development and team-based coding to create themes related to values of service learning. Creating a coding framework, authors created broad “parent” codes to identify general themes within interview transcripts and associated “child” codes for specificity within general themes; data were analyzed thematically to capture the nature of participants’ community-engaged experiences and what they gleaned from them. Through an inductive approach, team members independently coded interviews for preliminary data analysis, where codes were identified and extracted. Following this initial coding, we completed another round of coding with previously coded interviews to incorporate differing perspectives. Through the iterative codebook development process of the text, new child codes with more specific and nuanced subcategories were created accordingly as recurring themes emerged. After preliminary rounds of coding, the team reconciled codes in all interviews through rounds of meetings, which consisted of discussing, modifying, and agreeing upon codes in unanimous consensus. The research team determined that data sufficiency was reached when collected data adequately addressed the research objectives and provided comprehensive insights into the students’ attitudes toward medical service learning. Furthermore, the hour length and subsequent depth of each interview yielded rich data for analysis. A codebook was created which includes a written description for each child code that accurately represents the identified text. The reliability, validity, and generalizability of the study were ensured through multiple methods. The reliability of the qualitative data collection and analysis was ensured by following Silverman’s five approaches to reliability [34]. Additionally, validity was established through our deliberate sampling of participants who fit within the criteria of the study and could give insight into our framework through qualitative interviews. In addition, before results were finalized, member-checking was utilized by sending participants our coded themes, giving them an opportunity to provide any feedback to establish a more credible analysis.

## Results

### Quantitative findings

We received 9 completed CEQs from the 30 individuals who completed a course project from Autumn 2020 to Winter 2022 (30% response rate). Respondents include 5 (55%) males and 4 (45%) females. 6 (67%) of the respondents were undergraduate students and 3 (33%) were graduate students.

Internal consistency of the scales as measured by the Cronbach alpha coefficient was considered to be adequate (0.76–0.92). Mean scores of 4.0 on four of the five scales (good teaching, clear goals and standards, graduate qualities, and overall satisfaction) support this finding (Table 3). “Good teaching” received the highest mean score (4.65) while “generic skills” received the lowest mean score (3.87). Respondents reported that “instructors worked hard to make the course content interesting” (mean = 4.89), “instructors made it clear right from the start what they expected from students” (mean = 4.89), and “instructors normally gave me helpful feedback on how I was doing” (mean = 4.78), respectively, to be the top three strengths of the course. While no areas of dissatisfaction (score ≤ 3) were reported, the lowest scale was the “generic skills” scale (mean = 3.87). This was supported by the fact that the four lowest scores reported in the CEQ belong to the “generic skills” scale (Table 4).

**Table 4:** Course experience questionnaire scale and mean response of each item (*n* = 9)

Item	Mean	SD	Scale	Question
1	4.67	0.5	Good teaching	The instructors put a lot of time into commenting on my work.
2	4.78	0.44	Good teaching	The instructors normally gave me helpful feedback on how I was doing.
3	3.33	1.32	Generic skills	The course helped me develop my ability to work as a team member.
4	4.22	1.09	Clear goals and standards	It was always easy to know the standard of work expected in this course.
5	4.56	0.88	Good teaching	The instructors of this course motivated me to do my best work.
6	4	1.22	Graduate qualities	The course provided me with a broad overview of my field of knowledge.
7	3.56	1.13	Generic skills	The course sharpened my analytic skills.
8	4.33	0.71	Good teaching	The instructors were extremely good at explaining things in this course.
9	4.89	0.33	Good teaching	The instructors worked hard to make the course content interesting.
10	4.44	1.01	Graduate qualities	The course developed my confidence to investigate new ideas.
11	4.44	1.01	Generic skills	The course developed my problem-solving skills.
12	4.67	0.71	Good teaching	The instructors made a real effort to understand the difficulties I might be having with my coursework.
13	4.11	1.45	Clear goals and standards	I usually had a clear idea of where I was going and what was expected of me in this course.
14	4.67	0.71	Graduate qualities	The course stimulated my enthusiasm for further learning.
15	3.89	0.93	Generic skills	The course improved my skills in written communication.
16	4.11	1.17	Graduate qualities	I learned to apply principles from this course to new situations.
17	4.67	0.71	Graduate qualities	I consider what I learned in this course valuable for my future.
18	3.89	1.27	Generic skills	As a result of this course, I feel confident about tackling unfamiliar problems.
19	4.11	0.78	Generic skills	This course helped me to develop the ability to plan my own work.
20	4.89	0.33	Clear goals and standards	The instructors made it clear right from the start what they expected from students.
21	3.89	1.27	Generic skills	The course helped me develop my oral presentation skills.
22	4.44	0.88	Graduate qualities	The overall course experience encouraged me to value perspectives other than my own.
23	4.56	0.73	Overall satisfaction	Overall, I was satisfied with the quality of this course.

* *Note* Responses were 5-point options, from 1 (‘‘strongly disagree’’) to 5 (‘‘strongly agree’’)

### Qualitative findings

Five of the nine students who completed the CEQ were interviewed. A total of three major themes emerged from the data: Multi-Perspective Project Feedback Enhances Students’ Autonomous, Community-Engaged Experiences, Students’ Community-Engaged Course Experiences Encourage Relatedness and Collaboration, and Students Indicate Empowerment for Future Medical Service Work. Each provided further insight into how the course was able to further students’ academic and professional goals and help them create sustainable community collaborations, as demonstrated through SDT (Table 4). Below we describe each theme, followed by illustrative quotes from interviews pertaining to each theme. “P” has been used to indicate participants, followed by their associated number.

### Multi-perspective project feedback enhances students’ autonomous, community-engaged experiences

The first theme refers to the autonomous nature of students’ community health interventions, and how multi-perspective feedback was provided to best support this autonomy (Table 5). Students detailed how instructor, community partner, and peer feedback was beneficial to their community health interventions. Furthermore, students described how the versatile nature of instructor feedback helped their projects grow, even with the interdisciplinary variety of student projects. For example, P2 describes “how versatile the teaching assistants were because there were a wide range of different projects in the course, and they were able to offer good, substantial feedback for all of them, and so I think that the mentorship also really helped me improve and develop my project.” The helpful nature of feedback was supported by community partners’ feedback. Students gained a unique understanding of the communities with which they worked and felt increasingly competent as a result of this understanding. For example, P5 said:

I know more about [Community Partner’s] terminologies and how they deal with problems, or why that does not work well in real-world practice and what difficulties they face. Those are very valuable information I could not find through papers, publications, social media, or news articles.

**Table 5:** Representative evidence for each theme and subtheme representative evidence for each theme and subtheme

Theme and subthemes	Qualitative evidence	Quantitative evidence
Multi-perspective feedback		
*Instructor Feedback*	“I liked how versatile the TAs were because there was a wide range of different projects in the class and they were able to offer good, substantial feedback for all of them, and so I think that the mentorship also really helped me improve and develop my project.” **(P2)**	**Good Teaching Scale** “The teaching staff normally gave me helpful feedback on how I was going.” (mean = 4.78) “The staff put a lot of time into commenting on my work.” (mean = 4.67)
*Community Partner Feedback*	“A lot of the changes I made throughout my project were based on direct feedback from different parts of the community, it was […] a collaboration between me and the community.” **(P2)** “I know more about [Community Partner’s] terminologies and how they deal with problems, or why that does not work well in real-world practice and what difficulties they face. Those are very valuable information I could not find through papers, publications, social media, or news articles.” **(P5)**	**Good Teaching Scale** “The staff put a lot of time into commenting on my work.” (mean = 4.67)
*Peer Feedback*	“I really did appreciate how much we took [other students’] input and the space we were allowed to do that.” **(P1)**	
**Encouraging Collaboration**		
*Interdisciplinary Partnerships*	“Through this course, I was able to contact and interview several practitioners in disaster relief or COVID-19. I think this course strengthened my mindset or the idea that it’s not always hard to reach out to practitioners in the industry or in the frontline and integrate real-world policy into my research.” **(P5)** **“**What I’m really proud of was the ability that I had to bring so many different experts together […] it was the result of communicating with so many different experts to get their input on what is the best way to make this communication and looking at the research to see what’s the best way to communicate about vaccines and bringing all that together, because I think that is sort of the lesson of the class: how can we create a project that’s really interdisciplinary.” **(P1)**	**Generic Skills Scale** “My university experience encouraged me to value perspectives other than my own.” (mean = 4.44) **Generic Skills Scale** “The course helped me develop my ability as a team member.” (mean = 3.33)
*Long Lasting Connections*	“It was invaluable to have the course at that time in my career […] it built my network in the community. These contacts, they’re not going away - I can contact people, five years later, and remember that experience.” **(P3)**	**Graduate Qualities Scale** “I consider what I learned valuable for my future.” (mean = 4.67)
**Empowerment for Future Community Engagement**		
*Affirming Choice in Medicine*	“Before [taking the course], I thought medicine made sense with my talents […] and that things that I see in my future career, but after doing this project, I saw it as a way I could be happy, like being someone who just works on [vaccine hesitancy] for a long time, makes that an important part of their career” **(P1)**	**Graduate Qualities Scale** “University stimulated my enthusiasm for further learning.” (mean = 4.67)
*Future Coursework*	“I took another community-engaged learning course […] I think that taking the COVID Elective course allowed me to more easily think: ‘Oh, I can do this; this is something I want to do’, and so that’s why I think this class was my gateway into more community-engaged learning.” **(P2)**	**Graduate Qualities Scale** “University stimulated my enthusiasm for further learning.” (mean = 4.67)
*Leadership*	“As future healthcare providers, we want to be leaders in the communities that we serve, and you want to be able to be involved at any time with the people of our community. This class made me realize that it’s important for us to have that experience.” **(P4)**	**Graduate Qualities Scale** “My university experience encouraged me to value perspectives other than my own.” (mean = 4.44)
*Sustainability*	“I have been able to continue working on creating content to combat vaccine hesitancy and make a couple […] of read-aloud videos of the book to further distribute the message of the book […] Since the class ended, I’ve done a few workshops in elementary schools […] teach about vaccines and I have another one coming up with Santa Clara Family Health Plan. I’m finishing up working on a lesson plan to accompany the book.” **(P1)**	**Graduate Qualities Scale** “University stimulated my enthusiasm for further learning.” (mean = 4.67)

Students appreciated that by co-developing projects with community partners, the utility and practicality of their proposed intervention were maximized. Likewise, students appreciated that they were able to glean feedback from their peers on their interventions. P1 mentioned how they “did appreciate how much we took [other students’] input and the space we were allowed to do that.” Consequently, the course created opportunities for students to receive multi-perspective feedback that enabled them to autonomously pursue health interventions, increasing their motivation to engage in their chosen project and community.

### Students’ community-engaged experiences encourage relatedness and collaboration

The second theme encompasses how the course encouraged students to co-develop projects community partners, gaining relatedness and feelings of competence in desired fields. In doing so, students were motivated to nurture both interdisciplinary partnerships and long-lasting connections that continued beyond the course. Students describe being able to connect and collaborate with an interdisciplinary group of community partners to create projects together, and how they learned valuable lessons from the experience. For example, P5 describes how they were “able to contact and interview several practitioners in disaster relief or COVID-19. I think this course strengthened my mindset or the idea that it’s not always hard to reach out to practitioners in the industry or in the frontline and integrate real-world policy into my research.” P1 also touches on their interdisciplinary partnerships:

What I’m really proud of was the ability that I had to bring so many different experts together […] it was the result of communicating with so many different experts to get their input on what is the best way to make this communication and looking at the research to see what’s the best way to communicate about vaccines and bringing all that together, because I think that is sort of the lesson of the class: how can we create a project that’s really interdisciplinary.

In creating these community partnerships, students were able to create long-lasting connections that expanded their network even after their participation in the course ended. For example, P3 describes:

It was invaluable to have the course at that time in my career […] it built my network in the community. These contacts, they’re not going away - I can contact people, five years later, and remember that experience.

Hence, the course helped nurture interdisciplinary, long-lasting community partnerships with students; the relatedness they gained from the course led to motivation to continue fostering their community partnerships well beyond the curriculum.

### Students indicate empowerment for future medical service work

The third theme details how the course motivated students to continue pursuing medical service work through their increased autonomy, competence, and relatedness. Students describe how the course affirmed their desire to pursue medicine, encouraged future community-engaged coursework, developed their leadership skills, and created sustainable projects that continued beyond the scope of the course. For some students, the nature of this course affirmed their choice in medicine and the potential for medicine to bring them fulfillment. For example, P1 describes:

Before [taking the course], I thought medicine made sense with my talents […] and that things that I see in my future career, but after doing this project, I saw it as a way I could be happy, like being someone who just works on [vaccine hesitancy] for a long time, makes that an important part of their career.

Furthermore, students describe how this course encouraged them to further pursue community-engaged work in academic contexts. For example, P2 mentioned how they “took another community-engaged course”, and that taking this course “allowed me to more easily think: ‘Oh, I can do this; this is something I want to do’, and so that’s why I think this class was my gateway into more community-engaged learning.” In addition, this course allowed students to realize the importance of community-engaged learning, especially in the field of healthcare. For example, P4 says:

As future healthcare providers, we want to be leaders in the communities that we serve, and you want to be able to be involved at any time with the people of our community. This class made me realize that it’s important for us to have that experience.

In addition, students were able to create sustainable community health interventions that they pursued even after their involvement with the course. For example, P1 details:

I have been able to continue working on creating content to combat vaccine hesitancy and make a couple […] of read-aloud videos of the book to further distribute the message of the book […] Since the class ended, I’ve done a few workshops in elementary schools […] teach about vaccines and I have another one coming up with Santa Clara Family Health Plan. I’m finishing up working on a lesson plan to accompany the book.

Thus, this course became an important catalyst in motivating students for future community engagement, whether it be through pursuing future coursework, affirming a medical career interest, creating sustainable health interventions, or a developing heightened sense of leadership.

## Discussion

**Fig. 1:**
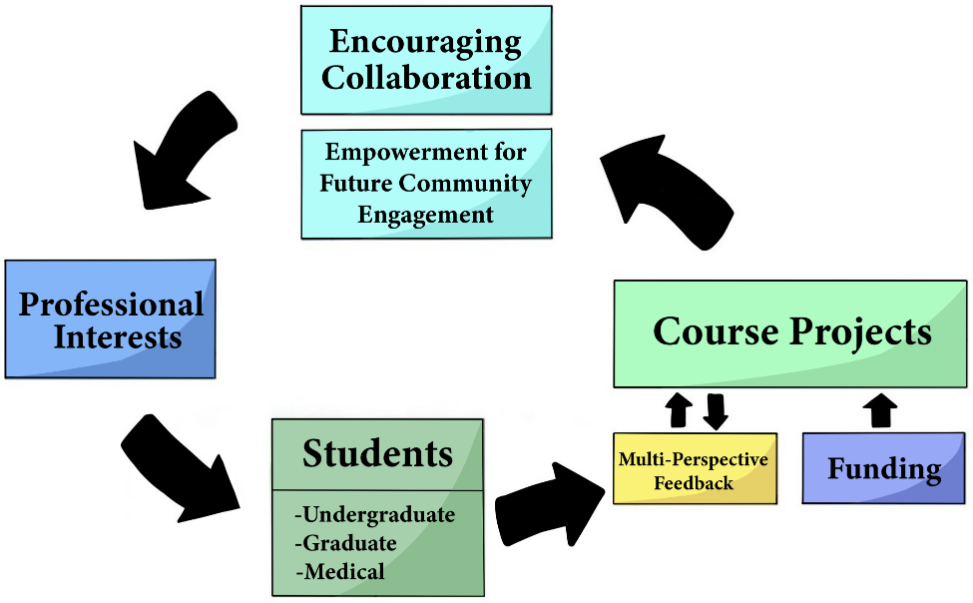
Our Curriculum Visualized. This process diagram represents the chronological flowchart for students taking the course, and how the course supports their community-engaged learning. This figure was created using adobe photoshop and clip studio paint

In this mixed-methods study, we evaluated students’ attitudes toward a medical service learning course and its ability to support students in designing and implementing community interventions. The course not only provided students with professionally-relevant experiential learning, but also affirmed their intended medical career and professional interests and inspired confidence in navigating future projects (Fig. 1). The course poised students to form meaningful relationships with communities and professionals in their field of interest via direct introduction to such community members, further inspiring their professional interests and disciplines. Students described the desire to continue their projects and community relationships after finishing the course, suggesting that the course helped students realize sustainable and impactful projects. These community partnerships and empowerment for future community engagement were possible due to the autonomy that the students were given through this course, as they were able to self-design their health intervention and choose their own community to aid. Ultimately, this course may serve as a gateway course into medical service learning. The course incorporates community health work into students’ coursework and functions as a guide to any student interested in discovering how their interests intersect with community health (Fig. 1). In addition, these findings align with SDT, where students, upon taking this course, indicate higher levels of autonomy and competence in their community-engaged work and feel a greater level of relatedness to their community-engaged work, ultimately motivating them to pursue future community-engaged work.

While many service learning opportunities exist, our course affords students greater autonomy and agency in their volunteerism by virtue of a novel mentorship strategy, directly addressing how existing medical service learning limits student autonomy and capacity for leadership. In fact, affording students a greater degree of autonomy and control in creating their learning experience was one of the original impetus for service learning [35, 36]. The multi-perspective feedback provided by course instructors, community partners, and students’ peers was the key to project implementation, as supported by student testimonials and the CEQ. Hence, this layered system of project-specific feedback awarded students both the capacity to efficiently address a community need and the autonomy to self-design a project in a field of their choosing that they otherwise may not have been able to complete. By allowing students to self-design their own projects in fields of their interests, they experienced autonomy, which is a key factor in increased motivation in SDT. The Teaching Team was able to provide helpful feedback to all students pursuing projects, regardless of their specific field, allowing any student to pursue an intervention in their area of interest. In doing so, the course not only provides students with previously characterized benefits of medical service learning through student autonomy, such as increased cultural competency and interpersonal skills, but also better prepares students in any field to be active leaders in future community health projects. Not only do students feel more autonomy over their work, but the feedback the course gives increases their feelings of competence, which SDT determines is a framework for increased motivation in their education and work.

Therefore, students take on a more active role in addressing community needs, which is one of the most important differences between medical service learning and classroom learning [36]. Rather than filling pre-identified volunteer roles, as is often the norm in service learning, students forge their own paths. As a result, students are more personally invested in their projects and are motivated to undertake future community-engaged work, as supported by both the CEQ and student testimonials. Across all interviews, students demonstrated a sense of empowerment and agency, as their experience in the course allowed them to realize they are capable of driving community-engaged projects of their own; these feelings of competence are vital to their motivations to continue engaging with communities in their fields of interest. In addition, with this sense of agency and leadership, students felt inspired by more personal relationships with their chosen communities. Students often described continuing relationships well beyond the course. Hence, the close level of engagement with their work increases the level of relatedness they feel in their work, which is key to SDT, and is shown to heighten student motivation, so much so that they continue to engage in the work after the course has finished.

Another notable difference between our pedagogy and previous approaches to service learning is its ability to engage students across the educational continuum, from first-year undergraduate to medical students and Ph.D. candidates, rather than having a homogeneous cohort of students. While many of our medical service learning students aspire to become physicians, we also have many students who are interested in working on community health issues but do not intend to become medical professionals. This is in stark contrast to most medical service learning courses, which often limit enrollment to medical students. By virtue of this interdisciplinary cohort of students, students gain extensive experience in working with both medical and non-medical collaborators in the context of community health. Additionally, the course allows pre-medical undergraduate students to accrue meaningful community health experience early on in their medical careers, and give them the autonomy to lead their own efforts in community health. Hence, this course not only prioritizes medical students’ experiences in community health, but also invites students of any field to discover how they can foster partnerships with communities through their fields of interest. This course awards students the opportunity to explore intersections of community health and interdisciplinary fields, opening new possibilities for interdisciplinary service learning.

Our course also addresses several historic limitations of service learning by ensuring student autonomy and leadership in projects, allowing students to make connections with communities and better training students of any professional background for future service learning. Community partner input in project conceptualization ensured that real community needs were met through the co-development process of the projects [10]. This is an important facet of community-based participatory research, where community partners and researchers initiating community projects are regarded as equals. The structure of our course allowed students and community partners to have more opportunities to become equal beneficiaries of the project by working together throughout the progression of each project [10]. During interviews, students noted changes implemented as a direct result of community partner feedback, highlighting the importance of community trust and feedback through cultural competency and sensitivity. Hence, students were able to participate in community health work while developing skills for improved collaboration, interpersonal skills, and building empathy. Consequently, this course addresses key limitations of medical service learning and leads to higher levels of student motivation by increasing autonomy, competence, and relatedness.

### Limitations

While we are encouraged by the results of our study, there are several limitations worth noting. Due to the relatively small number of past students, institutional review board guidance preventing students from participating in educational research before final grades are posted, and students graduating, we had a small sample size and low response rate, which may represent a biased sample. Additionally, we were limited to three follow-up emails per our IRB protocol. This low sample size may limit the reproducibility of our results, including the Cronbach alpha scores. Despite the study’s small sample size, a rigorous approach to quantitative and qualitative data analysis, including triangulation of methods, member checks, utilizing multiple independent analysts, and consulting external qualitative and quantitative experts, allowed us to glean meaningful insights into medical service learning. Studies with small sample sizes have the potential to produce rich qualitative findings, as demonstrated in the rigor of our analysis [37]. Given the high degree of recurring themes that emerged during the qualitative arm of the study, we concluded that we reached theoretical sufficiency. Upon analyzing the first two interviews, no new themes emerged as the subsequent interviews were coded. To answer more detailed questions, such as the difference in course experience between undergraduate and graduate students, a larger sample of participants is required.

## Conclusion

The presented curriculum demonstrates the benefit of giving students expanded roles and freedoms in medical service learning under the close advisory of course instructors and community experts. By virtue of this expanded role, students are better prepared to serve as leaders in future community-engaged work, rather than filling the traditional pre-defined role of a volunteer. While our course deals with the ongoing SARS-CoV-2 pandemic, our approach can be tailored to combat any community health issue.

### Electronic supplementary material

Below is the link to the electronic supplementary material.

Supplementary Material 1

## Data Availability

All data generated or analyzed during this study are included in this published article [and its supplementary information files].
