# Supplementary material for: Training the next generation of community-engaged physicians: a mixed-methods evaluation of a novel course for medical service learning in the COVID-19 era
Source: BMC Med Educ. 2024 Apr 22;24:426. doi: 10.1186/s12909-024-05372-8 (PMC11034080; doi:10.1186/s12909-024-05372-8)
Supplement: Supplementary file 1 — Supplementary Material 1 [file 12909_2024_5372_MOESM1_ESM.docx]

**Supplementary Digital Appendix**

**Item 1:** 1-1 Student Participant Interview Questions

1. Why did you decide to take PEDS 220: COVID-19 Elective?
2. Describe your experience with the course’s mentorship model.
3. What was most helpful about the mentorship model? What can be improved?
4. To what extent were you satisfied with the Teaching Team’s level of involvement in your project? If not, how would you have preferred it to be different?
5. How did completing a mentored, community-engaged project change your philosophy of education?
6. Describe your career aspirations. Have they changed or evolved as a result of taking PEDS 220 and completing a community-engaged project?
7. Describe your educational aspirations. Have they changed or evolved as a result of taking PEDS 220 and completing a community-engaged project?
8. What were the biggest takeaways from your project?
9. How will completing a community-engaged and mentored project change how you will approach future scholarly projects?
10. How did your course project impact your career trajectory in the short term?
11. How did your course project impact your career trajectory in the long term?
12. What did you learn about mentorship from taking this class?
13. Did you find your community partnership to be effective in reaching the goals of your project?
14. Were you satisfied with your community partner’s level of involvement in your project? If not, how would you have preferred it to be different?
